# Supplementary material for: In vivo interference of pea aphid endosymbiont Buchnera groEL gene by synthetic peptide nucleic acids
Source: Sci Rep. 2024 Mar 5;14:5378. doi: 10.1038/s41598-024-55179-2 (PMC10912616; doi:10.1038/s41598-024-55179-2)
Supplement: Supplementary file 1 — Supplementary Information 1. [file 41598_2024_55179_MOESM1_ESM.pdf]

[FindSequence.py]

```
import multiprocessing as mp
from functools import partial
from Bio import SeqIO
from Bio.SeqFeature import FeatureLocation
from Bio.Seq import reverse_complement


def find_sequence(genome_file, sequence, gff_file,num_processes=10):
    # Load genome sequences
    genome_records = list(SeqIO.parse(genome_file, "fasta"))

    # Create a dictionary of CDS and exon locations keyed by start
and end positions
    cds_dict = {}
    with open(gff_file, "r") as f:
        for line in f:
            if not line.startswith("#"):
                cols = line.strip().split("\t")
                feature = cols[2]
                if feature == "CDS" or feature == "exon":
                    start, end = map(int, cols[3:5])
                    strand = cols[6]
                    gene_name = cols[8].split(";")[2]+ "_" +
cols[8].split(";")[3] #gene_name = cols[8].split(";")[0].split("=")
[1]
                    if feature != "region":
                        cds_dict[(start, end, cols[0])] = (strand,
gene_name)

    # Find the locations of the query sequence
    query_length = len(sequence)
    locations = []
    for genome_record in genome_records:
        genome_seq = genome_record.seq
        chromosome_name = genome_record.id
        for strand, nucleotide_seq in [(+1, sequence), (-1,
reverse_complement(sequence))]:
            for i in range(len(genome_seq) - query_length + 1):
                num_matching_bases = sum([genome_seq[i+j].upper() ==
nucleotide_seq[j].upper() for j in range(query_length)])
                if num_matching_bases == query_length:
                    start_pos = i+1
                    end_pos = i+query_length
                    locations.append((chromosome_name, start_pos,
end_pos, strand, nucleotide_seq))

    # Find the CDSs that overlap with the query sequence
    overlapping_cds = []
    for cds_start, cds_end, cds_chromosome in cds_dict.keys():
        for query_chromosome, query_start, query_end, query_strand,
query_seq in locations:
            if cds_start <= query_end and cds_end >= query_start and
query_chromosome == cds_chromosome and cds_dict[(cds_start, cds_end,
cds_chromosome)][1] != "region":
```

```
                if query_strand == 1:
                    if cds_dict[(cds_start, cds_end,
cds_chromosome)][0] == "+":
                        strand_comment = "plus plus match"
                    else:
                        strand_comment = "reverse complement match"
                else:
                    if cds_dict[(cds_start, cds_end,
cds_chromosome)][0] == "+":
                        strand_comment = "reverse complement match"
                    else:
                        strand_comment = "plus plus match"
                cds_comment = ""
                if query_start <= cds_start <= query_end or
query_start <= cds_end <= query_end:
                    cds_comment = "Translational Start Site
Included"
                overlapping_cds.append((cds_dict[(cds_start,
cds_end, cds_chromosome)][1], cds_start, cds_end,
cds_dict[(cds_start, cds_end, cds_chromosome)][0], query_chromosome,
query_start, query_end, query_strand, strand_comment, cds_comment,
query_seq))



    return overlapping_cds


# Set the filenames and query sequence
#genome_file = "/home/kathrine/Apisum_Buchnera_genome_gff/
GCF_005508785.2_pea_aphid_22Mar2018_4r6ur_v2_genomic.fna" ##AL4f
#gff_file = "/home/kathrine/Apisum_Buchnera_genome_gff/
GCF_005508785.2_pea_aphid_22Mar2018_4r6ur_v2_genomic.gff"
#sequence = "GCCATTTGAC"

# Set the filenames and query sequence
genome_file = "/home/kathrine/Apisum_Buchnera_genome_gff/
GCF_000142985.2_Acyr_2.0_genomic.fna" ##LSR1
gff_file = "/home/kathrine/Apisum_Buchnera_genome_gff/genomic.gff"
sequence = "GCCATTTGAC"

# Set the filenames and query sequence
#genome_file = "/home/kathrine/Apisum_Buchnera_genome_gff/
GCF_000009605.1_ASM960v1_genomic.fna" ##Buchnera
#gff_file = "/home/kathrine/Apisum_Buchnera_genome_gff/
GCF_000009605.1_ASM960v1_genomic.gff"
#sequence = "GCCATTTGAC"


def write_results_to_file(results, output_file):
    with open(output_file, "w") as f:
        f.write("Gene Name\tCDS start position\tCDS end
position\tStrand\tQuery start position\tQuery end position\tQuery
strand\tComment\tExtra Comment\tQuery sequence\n")
```

```
        for result in results:
            f.write("\t".join(map(str, result)) + "\n")

# Run the function and write the results to a tab-delimited file
output_file = "/home/kathrine/Apisum_Buchnera_genome_gff/
20230411_bias5_LSR1_GCCATTTGAC_output.txt"
results = find_sequence(genome_file, sequence, gff_file)
write_results_to_file(results, output_file)
```

Buchnera_GCCATTTGAC_GroEL_match

Gene Name	CDS start position	CDS end position	Strand	Genome	Query start position	Query end position	Query strand	Comment	Extra Comment	Query sequence
WP_009873980.1	18715	20361	+	NC_002528.1	18710	18719	-1	reverse strand match	Translational Start Site Included	GTCAAATGGC
WP_010896104.1	451384	453021	+	NC_002528.1	451856	451865	-1	reverse strand match		GTCAAATGGC

Buchnera_GCGATTTGTC_mm_match

Gene Name	CDS start position	CDS end position	Strand	Genome	Query start position	Query end position	Query strand	Comment	Extra Comment	Query sequence

A.pisum_GCCATTTGAC_GroEL_match

Gene Name	CDS start position	CDS end position	Strand	Genome	Query start position	Query end position	Query strand	Comment	Extra Comment	Query sequence
cds-XP_016658335.1	63725824	63726288	-	NC_042493.1	63726056	63726065	-1	plus plus match		GTCAAATGGC
cds-XP_029345510.1	6492980	6493219	+	NC_042495.1	6493087	6493096	-1	reverse strand match		GTCAAATGGC
exon-XM_016804473.2-9	29460614	29460850	-	NC_042494.1	29460806	29460815	1	reverse complement match		GCCATTTGAC
cds-XP_008187795.1	81559887	81560822	-	NC_042493.1	81560758	81560767	-1	plus plus match		GTCAAATGGC
exon-XM_008182385.3-1	120091980	120094290	+	NC_042493.1	120092707	120092716	1	plus plus match		GCCATTTGAC
exon-XM_008182385.3-1	120091980	120094290	+	NC_042493.1	120092791	120092800	1	plus plus match		GCCATTTGAC
exon-XM_001943429.5-1	68103038	68103527	-	NC_042493.1	68103398	68103407	-1	plus plus match		GTCAAATGGC
cds-XP_001944787.1	46528160	46528488	-	NC_042494.1	46528370	46528379	-1	plus plus match		GTCAAATGGC
cds-XP_008179310.1	34837755	34838044	+	NC_042493.1	34837780	34837789	1	plus plus match		GCCATTTGAC
exon-XM_029485261.1-1	81564254	81564572	-	NC_042493.1	81564437	81564446	-1	plus plus match		GTCAAATGGC
exon-XM_003245688.4-6	44170109	44174560	-	NC_042495.1	44174054	44174063	-1	plus plus match		GTCAAATGGC
exon-XR_003839395.1-2	100096464	100110270	-	NC_042495.1	100107372	100107381	1	reverse complement match		GCCATTTGAC
exon-XM_008189898.3-1	122695443	122696589	+	NC_042493.1	122695729	122695738	1	plus plus match		GCCATTTGAC
exon-XM_001947555.5-16	118625202	118625375	+	NC_042493.1	118625227	118625236	-1	reverse strand match		GTCAAATGGC
cds-XP_029341117.1	79642038	79642148	-	NC_042493.1	79642070	79642079	-1	plus plus match		GTCAAATGGC
exon-XR_001679071.2-1	113175235	113175515	-	NC_042495.1	113175487	113175496	1	reverse complement match		GCCATTTGAC
exon-XM_003245260.4-3	53821162	53822212	+	NC_042495.1	53821927	53821936	1	plus plus match		GCCATTTGAC
exon-XM_003243785.4-2	69599513	69600683	+	NC_042495.1	69599927	69599936	1	plus plus match		GCCATTTGAC
cds-XP_029347159.1	16653447	16654306	-	NC_042496.1	16654219	16654228	-1	plus plus match		GTCAAATGGC
cds-XP_003242619.1	34654680	34654938	-	NC_042494.1	34654885	34654894	1	reverse complement match		GCCATTTGAC
cds-XP_029341121.1	81564254	81564501	-	NC_042493.1	81564437	81564446	-1	plus plus match		GTCAAATGGC
cds-XP_001947590.2	118625202	118625318	+	NC_042493.1	118625227	118625236	-1	reverse strand match		GTCAAATGGC
exon-XM_016802846.2-1	63725797	63726557	-	NC_042493.1	63726056	63726065	-1	plus plus match		GTCAAATGGC
cds-XP_008185766.1	15357619	15357751	-	NC_042493.1	15357672	15357681	-1	plus plus match		GTCAAATGGC
cds-XP_029341117.1	79647312	79647717	-	NC_042493.1	79647569	79647578	-1	plus plus match		GTCAAATGGC
exon-XM_029488183.1-1	64443188	64446047	-	NC_042493.1	64443794	64443803	-1	plus plus match		GTCAAATGGC
exon-XM_029488183.1-1	64443188	64446047	-	NC_042493.1	64444046	64444055	-1	plus plus match		GTCAAATGGC
exon-XM_029488183.1-1	64443188	64446047	-	NC_042493.1	64444382	64444391	-1	plus plus match		GTCAAATGGC
exon-XM_029488183.1-1	64443188	64446047	-	NC_042493.1	64444718	64444727	-1	plus plus match		GTCAAATGGC
exon-XM_029488183.1-1	64443188	64446047	-	NC_042493.1	64445054	64445063	-1	plus plus match		GTCAAATGGC
exon-XM_029488183.1-1	64443188	64446047	-	NC_042493.1	64445222	64445231	-1	plus plus match		GTCAAATGGC
cds-XP_008180596.1	120018756	120019638	-	NC_042493.1	120019039	120019048	-1	plus plus match		GTCAAATGGC
cds-XP_008180596.1	120018756	120019638	-	NC_042493.1	120019207	120019216	-1	plus plus match		GTCAAATGGC
exon-XM_003240907.4-2	79872191	79876470	+	NC_042495.1	79872371	79872380	1	plus plus match		GCCATTTGAC
exon-XM_001943281.5-2	5097667	5099026	-	NC_042495.1	5098401	5098410	-1	plus plus match		GTCAAATGGC
cds-XP_001944928.2	48265305	48265598	-	NC_042495.1	48265540	48265549	1	reverse complement match		GCCATTTGAC
cds-XP_008185205.1	39422717	39422867	+	NC_042494.1	39422812	39422821	-1	reverse strand match		GTCAAATGGC
cds-XP_008189623.1	39326600	39327459	-	NC_042493.1	39326791	39326800	1	reverse complement match		GCCATTTGAC
exon-XM_029492135.1-4	48178	48335	-	NW_02177034[illegible]	48301	48310	1	reverse complement match		GCCATTTGAC
cds-XP_016661870.1	4887912	4888127	+	NC_042494.1	4888084	4888093	-1	reverse strand match		GTCAAATGGC
cds-XP_001950205.1	95446431	95446638	+	NC_042495.1	95446438	95446447	1	plus plus match		GCCATTTGAC

A.pisum_GCCATTTGAC_GroEL_match

Gene Name	CDS start position	CDS end position	Strand	Genome	Query start position	Query end position	Query strand	Comment	Extra Comment	Query sequence
cds-XP_001945772.2	83005476	83005614	+	NC_042494.1	83005584	83005593	-1	reverse strand match		GTCAAATGGC
cds-XP_029347703.1	692	981	-	NW_02175932[illegible]	947	956	-1	plus plus match		GTCAAATGGC
exon-XM_001947498.5-1	53607411	53608496	+	NC_042493.1	53607565	53607574	1	plus plus match		GCCATTTGAC
exon-XM_001949152.5-3	58065652	58066262	-	NC_042495.1	58066151	58066160	-1	plus plus match		GTCAAATGGC
exon-XM_029486126.1-1	166588411	166589457	-	NC_042494.1	166589157	166589166	1	reverse complement match		GCCATTTGAC
exon-XR_001679551.1-1	131225199	131225675	+	NC_042493.1	131225455	131225464	1	plus plus match		GCCATTTGAC
exon-XM_008189573.3-1	81559762	81560944	-	NC_042493.1	81560758	81560767	-1	plus plus match		GTCAAATGGC
exon-XM_001947461.5-9	29460419	29460850	-	NC_042494.1	29460806	29460815	1	reverse complement match		GCCATTTGAC
exon-XM_001946477.5-6	71662333	71662639	+	NC_042495.1	71662400	71662409	-1	reverse strand match		GTCAAATGGC
exon-XM_008189490.3-1	169913348	169915422	+	NC_042494.1	169915033	169915042	1	plus plus match		GCCATTTGAC
cds-XP_008183216.1	42342111	42343745	-	NC_042493.1	42342988	42342997	-1	plus plus match		GTCAAATGGC
cds-XP_029346638.1	63273602	63274093	+	NC_042495.1	63273677	63273686	-1	reverse strand match		GTCAAATGGC
cds-XP_029344043.1	64443679	64445370	-	NC_042493.1	64443794	64443803	-1	plus plus match		GTCAAATGGC
cds-XP_029344043.1	64443679	64445370	-	NC_042493.1	64444046	64444055	-1	plus plus match		GTCAAATGGC
cds-XP_029344043.1	64443679	64445370	-	NC_042493.1	64444382	64444391	-1	plus plus match		GTCAAATGGC
cds-XP_029344043.1	64443679	64445370	-	NC_042493.1	64444718	64444727	-1	plus plus match		GTCAAATGGC
cds-XP_029344043.1	64443679	64445370	-	NC_042493.1	64445054	64445063	-1	plus plus match		GTCAAATGGC
cds-XP_029344043.1	64443679	64445370	-	NC_042493.1	64445222	64445231	-1	plus plus match		GTCAAATGGC
cds-XP_001952865.2	26600429	26600868	-	NC_042496.1	26600583	26600592	1	reverse complement match		GCCATTTGAC
cds-XP_008180597.1	119892044	119893651	-	NC_042493.1	119892159	119892168	-1	plus plus match		GTCAAATGGC
cds-XP_008180597.1	119892044	119893651	-	NC_042493.1	119892243	119892252	-1	plus plus match		GTCAAATGGC
cds-XP_008180597.1	119892044	119893651	-	NC_042493.1	119892831	119892840	-1	plus plus match		GTCAAATGGC
cds-XP_008180597.1	119892044	119893651	-	NC_042493.1	119893251	119893260	-1	plus plus match		GTCAAATGGC
exon-XM_029491843.1-4	573	981	-	NW_02175932[illegible]	947	956	-1	plus plus match		GTCAAATGGC
cds-XP_029344270.1	9451389	9451606	-	NC_042494.1	9451578	9451587	1	reverse complement match		GCCATTTGAC
cds-XP_001950004.1	62072781	62073006	-	NC_042493.1	62072801	62072810	-1	plus plus match		GTCAAATGGC
cds-XP_016658319.1	120069256	120070567	+	NC_042493.1	120070067	120070076	1	plus plus match		GCCATTTGAC
exon-XM_008182374.3-3	120018677	120019638	-	NC_042493.1	120019039	120019048	-1	plus plus match		GTCAAATGGC
exon-XM_008182374.3-3	120018677	120019638	-	NC_042493.1	120019207	120019216	-1	plus plus match		GTCAAATGGC
cds-XP_029342462.1	9505408	9505625	-	NC_042494.1	9505597	9505606	1	reverse complement match		GCCATTTGAC
cds-XP_008183161.1	40600887	40601048	+	NC_042494.1	40601019	40601028	-1	reverse strand match		GTCAAATGGC
cds-XP_016663536.2	130459746	130463360	-	NC_042494.1	130459945	130459954	-1	plus plus match		GTCAAATGGC
cds-XP_016663536.2	130459746	130463360	-	NC_042494.1	130460533	130460542	-1	plus plus match		GTCAAATGGC
cds-XP_016663536.2	130459746	130463360	-	NC_042494.1	130463053	130463062	-1	plus plus match		GTCAAATGGC
exon-XR_003840300.1-1	132134876	132136216	-	NC_042493.1	132135929	132135938	1	reverse complement match		GCCATTTGAC
exon-XM_029486128.1-1	166588411	166589465	-	NC_042494.1	166589157	166589166	1	reverse complement match		GCCATTTGAC
exon-XM_029486127.1-1	166588605	166589457	-	NC_042494.1	166589157	166589166	1	reverse complement match		GCCATTTGAC
cds-XP_003246532.1	136048986	136049233	-	NC_042494.1	136049117	136049126	1	reverse complement match		GCCATTTGAC
exon-XM_001951318.5-10	31518423	31519855	+	NC_042493.1	31519395	31519404	1	plus plus match		GCCATTTGAC
cds-XP_029347018.1	31352993	31353259	+	NC_042496.1	31353011	31353020	1	plus plus match		GCCATTTGAC
exon-XM_029489515.1-2	76869587	76870034	+	NC_042493.1	76869680	76869689	-1	reverse strand match		GTCAAATGGC

A.pisum_GCCATTTGAC_GroEL_match

Gene Name	CDS start position	CDS end position	Strand	Genome	Query start position	Query end position	Query strand	Comment	Extra Comment	Query sequence
cds-XP_008180149.1	35683480	35685756	+	NC_042493.1	35685544	35685553	-1	reverse strand match		GTCAAATGGC
cds-NP_001313587.1	32809694	32810075	+	NC_042496.1	32810054	32810063	-1	reverse strand match		GTCAAATGGC
cds-XP_003245736.1	44171543	44174560	-	NC_042495.1	44174054	44174063	-1	plus plus match		GTCAAATGGC
exon-XM_016802832.2-1	120077075	120078543	+	NC_042493.1	120077488	120077497	1	plus plus match		GCCATTTGAC
exon-XM_016802832.2-1	120077075	120078543	+	NC_042493.1	120077740	120077749	1	plus plus match		GCCATTTGAC
exon-XM_016802832.2-1	120077075	120078543	+	NC_042493.1	120078076	120078085	1	plus plus match		GCCATTTGAC
exon-XM_029489508.1-2	76869587	76869803	+	NC_042493.1	76869680	76869689	-1	reverse strand match		GTCAAATGGC
id-LOC100575151	120112761	120114036	-	NC_042493.1	120113072	120113081	-1	plus plus match		GTCAAATGGC
id-LOC100575151	120112761	120114036	-	NC_042493.1	120113408	120113417	-1	plus plus match		GTCAAATGGC
id-LOC100575151	120112761	120114036	-	NC_042493.1	120113660	120113669	-1	plus plus match		GTCAAATGGC
exon-XM_003245114.4-1	19848840	19849098	-	NC_042494.1	19849091	19849100	1	reverse complement match	Translational Start Site Included	GCCATTTGAC
cds-XP_008190068.1	165722005	165722159	+	NC_042494.1	165722064	165722073	-1	reverse strand match		GTCAAATGGC
cds-XP_029341345.1	123253114	123254319	+	NC_042493.1	123253301	123253310	1	plus plus match		GCCATTTGAC
cds-XP_029341345.1	123253114	123254319	+	NC_042493.1	123253469	123253478	1	plus plus match		GCCATTTGAC
cds-XP_029341345.1	123253114	123254319	+	NC_042493.1	123253973	123253982	1	plus plus match		GCCATTTGAC
cds-XP_029341345.1	123253114	123254319	+	NC_042493.1	123254141	123254150	1	plus plus match		GCCATTTGAC
cds-XP_001950350.1	28975667	28975817	-	NC_042493.1	28975713	28975722	1	reverse complement match		GCCATTTGAC
cds-XP_016662530.1	67732156	67732399	+	NC_042495.1	67732323	67732332	1	plus plus match		GCCATTTGAC
cds-XP_001943316.2	5098398	5099026	-	NC_042495.1	5098401	5098410	-1	plus plus match		GTCAAATGGC
exon-XM_008182375.3-1	119891862	119893693	-	NC_042493.1	119892159	119892168	-1	plus plus match		GTCAAATGGC
exon-XM_008182375.3-1	119891862	119893693	-	NC_042493.1	119892243	119892252	-1	plus plus match		GTCAAATGGC
exon-XM_008182375.3-1	119891862	119893693	-	NC_042493.1	119892831	119892840	-1	plus plus match		GTCAAATGGC
exon-XM_008182375.3-1	119891862	119893693	-	NC_042493.1	119893251	119893260	-1	plus plus match		GTCAAATGGC
cds-XP_029341509.1	62498821	62499336	+	NC_042493.1	62499044	62499053	1	plus plus match		GCCATTTGAC
exon-XM_001944752.5-6	46526996	46528488	-	NC_042494.1	46528370	46528379	-1	plus plus match		GTCAAATGGC
cds-NP_001153860.1	53416086	53416278	+	NC_042494.1	53416103	53416112	-1	reverse strand match		GTCAAATGGC
cds-XP_008180607.1	120091980	120094007	+	NC_042493.1	120092707	120092716	1	plus plus match		GCCATTTGAC
cds-XP_008180607.1	120091980	120094007	+	NC_042493.1	120092791	120092800	1	plus plus match		GCCATTTGAC
cds-XP_016658321.1	120077097	120078452	+	NC_042493.1	120077488	120077497	1	plus plus match		GCCATTTGAC
cds-XP_016658321.1	120077097	120078452	+	NC_042493.1	120077740	120077749	1	plus plus match		GCCATTTGAC
cds-XP_016658321.1	120077097	120078452	+	NC_042493.1	120078076	120078085	1	plus plus match		GCCATTTGAC
cds-XP_008185369.1	109228636	109228776	+	NC_042494.1	109228712	109228721	1	plus plus match		GCCATTTGAC
exon-XM_003242470.4-1	127610202	127610589	+	NC_042493.1	127610460	127610469	1	plus plus match		GCCATTTGAC
exon-XM_016807794.2-3	22272260	22272603	+	NC_042494.1	22272480	22272489	-1	reverse strand match		GTCAAATGGC
cds-XP_008188120.1	122695590	122696441	+	NC_042493.1	122695729	122695738	1	plus plus match		GCCATTTGAC
cds-XP_016663283.1	22272276	22272603	+	NC_042494.1	22272480	22272489	-1	reverse strand match		GTCAAATGGC
exon-XM_029485257.1-1	79647312	79647778	-	NC_042493.1	79647569	79647578	-1	plus plus match		GTCAAATGGC
exon-XM_029486931.1-17	32710988	32714100	-	NC_042494.1	32711640	32711649	1	reverse complement match		GCCATTTGAC
cds-XP_029347864.1	18781090	18781305	-	NC_042493.1	18781219	18781228	-1	plus plus match		GTCAAATGGC
exon-XM_008184994.3-1	42342014	42343778	-	NC_042493.1	42342988	42342997	-1	plus plus match		GTCAAATGGC

A.pisum_GCCATTTGAC_GroEL_match

Gene Name	CDS start position	CDS end position	Strand	Genome	Query start position	Query end position	Query strand	Comment	Extra Comment	Query sequence
exon-XM_003247813.3-10	31639514	31640930	+	NC_042493.1	31640478	31640487	1	plus plus match		GCCATTTGAC
cds-XP_029345615.1	78232162	78232311	+	NC_042495.1	78232273	78232282	-1	reverse strand match		GTCAAATGGC
cds-XP_001949187.1	58065931	58066262	-	NC_042495.1	58066151	58066160	-1	plus plus match		GTCAAATGGC
cds-XP_016659962.1	29461709	29461819	-	NC_042494.1	29461770	29461779	1	reverse complement match		GCCATTTGAC
cds-XP_008180595.2	120153375	120154466	-	NC_042493.1	120153982	120153991	-1	plus plus match		GTCAAATGGC
cds-XP_008180595.2	120153375	120154466	-	NC_042493.1	120154150	120154159	-1	plus plus match		GTCAAATGGC
cds-XP_001948437.3	43302540	43303168	-	NC_042493.1	43302674	43302683	-1	plus plus match		GTCAAATGGC
exon-XM_008181088.3-6	34837755	34838080	+	NC_042493.1	34837780	34837789	1	plus plus match		GCCATTTGAC
cds-XP_003244911.1	44190258	44190553	+	NC_042494.1	44190514	44190523	1	plus plus match		GCCATTTGAC
cds-XP_001949364.2	83180546	83180650	-	NC_042494.1	83180617	83180626	1	reverse complement match		GCCATTTGAC
cds-XP_001948485.2	63703330	63704610	+	NC_042493.1	63704225	63704234	1	plus plus match		GCCATTTGAC
cds-XP_003240955.1	79872309	79875923	+	NC_042495.1	79872371	79872380	1	plus plus match		GCCATTTGAC
cds-XP_008180907.1	120364310	120366169	+	NC_042493.1	120364869	120364878	1	plus plus match		GCCATTTGAC
cds-XP_008180907.1	120364310	120366169	+	NC_042493.1	120365121	120365130	1	plus plus match		GCCATTTGAC
cds-XP_003243833.1	69599513	69600527	+	NC_042495.1	69599927	69599936	1	plus plus match		GCCATTTGAC
cds-XP_008180549.2	40083412	40083834	+	NC_042496.1	40083723	40083732	-1	reverse strand match		GTCAAATGGC
exon-XR_001679550.1-1	131225199	131225735	+	NC_042493.1	131225455	131225464	1	plus plus match		GCCATTTGAC
cds-XP_029341117.1	79641536	79641870	-	NC_042493.1	79641819	79641828	-1	plus plus match		GTCAAATGGC
exon-XM_008182327.3-1	40082444	40083834	+	NC_042496.1	40083723	40083732	-1	reverse strand match		GTCAAATGGC
exon-XM_001949329.5-1	83180546	83180752	-	NC_042494.1	83180617	83180626	1	reverse complement match		GCCATTTGAC
cds-XP_008190067.1	165721918	165722159	+	NC_042494.1	165722064	165722073	-1	reverse strand match		GTCAAATGGC
exon-XM_001948450.5-1	63702882	63704726	+	NC_042493.1	63704225	63704234	1	plus plus match		GCCATTTGAC
cds-XP_029347556.1	33663054	33663253	+	NC_042496.1	33663228	33663237	-1	reverse strand match		GTCAAATGGC
cds-XP_008183306.3	48398248	48398541	-	NC_042495.1	48398483	48398492	1	reverse complement match		GCCATTTGAC
cds-XP_003244828.1	14100096	14100267	-	NC_042495.1	14100179	14100188	1	reverse complement match		GCCATTTGAC
exon-XM_029491964.1-15	18728473	18729960	-	NC_042493.1	18729016	18729025	-1	plus plus match		GTCAAATGGC
cds-XP_016664264.1	16128258	16128369	-	NC_042493.1	16128271	16128280	1	reverse complement match		GCCATTTGAC
exon-XR_003839441.1-5	105687339	105692839	-	NC_042495.1	105692236	105692245	1	reverse complement match		GCCATTTGAC
cds-XP_016659962.1	29460725	29460850	-	NC_042494.1	29460806	29460815	1	reverse complement match		GCCATTTGAC
exon-NM_001326658.1-1	32809591	32810075	+	NC_042496.1	32810054	32810063	-1	reverse strand match		GTCAAATGGC
exon-XM_016808047.2-1	130458625	130463863	-	NC_042494.1	130459945	130459954	-1	plus plus match		GTCAAATGGC
exon-XM_016808047.2-1	130458625	130463863	-	NC_042494.1	130460533	130460542	-1	plus plus match		GTCAAATGGC
exon-XM_016808047.2-1	130458625	130463863	-	NC_042494.1	130463053	130463062	-1	plus plus match		GTCAAATGGC
exon-XM_029485649.1-1	62498675	62499495	+	NC_042493.1	62499044	62499053	1	plus plus match		GCCATTTGAC
cds-XP_001946512.2	71662333	71662491	+	NC_042495.1	71662400	71662409	-1	reverse strand match		GTCAAATGGC
exon-XM_016802830.2-1	120069245	120070567	+	NC_042493.1	120070067	120070076	1	plus plus match		GCCATTTGAC
cds-XP_003246086.1	109257969	109258074	-	NC_042494.1	109258010	109258019	-1	plus plus match		GTCAAATGGC
cds-XP_029341238.1	106608676	106608906	+	NC_042493.1	106608677	106608686	-1	reverse strand match		GTCAAATGGC

A.pisum_GCGATTTGTC_mm_match

Gene Name	CDS start position	CDS end position	Strand	Genome	Query start position	Query end position	Query strand	Comment	Extra Comment	Query sequence
Dbxref=GeneID:103308676,Genbank:XR_003839373.1_gbkey=misc_RNA	23502250	23503438	-	NC_042495.1	23503251	23503260	1	reverse complement match		GCGATTTGTC
Dbxref=GeneID:100166278,Genbank:XP_008181700.1,APHIDBASE:ACYPI007162_ Name=XP_008181700.1	10029538	10029654	-	NC_042494.1	10029622	10029631	-1	plus plus match		GACAAATCGC
Dbxref=GeneID:100573041,Genbank:XM_008183828.3_gbkey=mRNA	46328606	46330081	+	NC_042494.1	46329759	46329768	1	plus plus match		GCGATTTGTC
Dbxref=GeneID:100568756,Genbank:XM_003241628.4_gbkey=mRNA	109868848	109880057	-	NC_042494.1	109879646	109879655	-1	plus plus match		GACAAATCGC
Dbxref=GeneID:103308676,Genbank:XM_029489120.1_gbkey=mRNA	23500469	23503437	-	NC_042495.1	23501504	23501513	1	reverse complement match		GCGATTTGTC
Dbxref=GeneID:103308676,Genbank:XM_029489120.1_gbkey=mRNA	23500469	23503437	-	NC_042495.1	23503251	23503260	1	reverse complement match		GCGATTTGTC
Dbxref=GeneID:100166426,Genbank:XP_008187030.1,APHIDBASE:ACYPI007298_ Name=XP_008187030.1	51386010	51386547	+	NC_042494.1	51386201	51386210	1	plus plus match		GCGATTTGTC
Dbxref=GeneID:100167291,Genbank:XM_001950167.5,APHIDBASE:ACYPI008099_ gbkey=mRNA	113909748	113910398	-	NC_042495.1	113909907	113909916	-1	plus plus match		GACAAATCGC
Dbxref=GeneID:100167857,Genbank:XM_001943301.5,APHIDBASE:ACYPI008613_ gbkey=mRNA	26005501	26006167	-	NC_042493.1	26005759	26005768	-1	plus plus match		GACAAATCGC
Dbxref=GeneID:100572049,Genbank:XP_029342142.1_Name=XP_029342142.1	42628823	42629003	+	NC_042493.1	42628864	42628873	1	plus plus match		GCGATTTGTC
Dbxref=GeneID:100573273,Genbank:XM_008186679.3_gbkey=mRNA	95964187	95964500	-	NC_042495.1	95964381	95964390	1	reverse complement match		GCGATTTGTC
Dbxref=GeneID:100161902,Genbank:XP_029343110.1,APHIDBASE:ACYPI003090_ Name=XP_029343110.1	51981324	51981500	+	NC_042494.1	51981439	51981448	-1	reverse strand match		GACAAATCGC
Dbxref=GeneID:107882420,Genbank:XR_001678734.2_gbkey=ncRNA	41653564	41656256	+	NC_042495.1	41653646	41653655	1	plus plus match		GCGATTTGTC
Dbxref=GeneID:100570516,Genbank:XM_003241639.4_gbkey=mRNA	18666249	18666792	-	NC_042494.1	18666485	18666494	-1	plus plus match		GACAAATCGC
Dbxref=GeneID:100574667,Genbank:XP_003242344.1_Name=XP_003242344.1	92459407	92459585	-	NC_042493.1	92459455	92459464	1	reverse complement match		GCGATTTGTC
Dbxref=GeneID:100573273,Genbank:XM_008186677.3_gbkey=mRNA	95964179	95964500	-	NC_042495.1	95964381	95964390	1	reverse complement match		GCGATTTGTC
Dbxref=GeneID:100574667,Genbank:XP_029347125.1_Name=XP_029347125.1	92372640	92372818	-	NC_042493.1	92372688	92372697	1	reverse complement match		GCGATTTGTC
Dbxref=GeneID:103311695,Genbank:XM_029491900.1_gbkey=mRNA	344	495	+	NW_021763465.1	448	457	-1	reverse strand match		GACAAATCGC
Dbxref=GeneID:103312012,Genbank:XP_008190208.1_Name=XP_008190208.1	12612188	12612441	-	NC_042496.1	12612363	12612372	-1	plus plus match		GACAAATCGC
Dbxref=GeneID:100160560,Genbank:XP_001951146.2,APHIDBASE:ACYPI001848_ Name=XP_001951146.2	41399224	41399341	+	NC_042494.1	41399243	41399252	1	plus plus match		GCGATTTGTC
Dbxref=GeneID:115034238,Genbank:XM_029490409.1_gbkey=mRNA	42648124	42649895	+	NC_042495.1	42648599	42648608	-1	reverse strand match		GACAAATCGC
Dbxref=GeneID:100573273,Genbank:XM_008186680.3_gbkey=mRNA	95964089	95964500	-	NC_042495.1	95964381	95964390	1	reverse complement match		GCGATTTGTC
Dbxref=GeneID:100169447,Genbank:XP_008185336.1,APHIDBASE:ACYPI010063_ Name=XP_008185336.1	25633998	25634104	+	NC_042496.1	25634017	25634026	-1	reverse strand match		GACAAATCGC
Dbxref=GeneID:103308676,Genbank:XR_003839371.1_gbkey=misc_RNA	23500469	23501572	-	NC_042495.1	23501504	23501513	1	reverse complement match		GCGATTTGTC
Dbxref=GeneID:100165198,Genbank:XP_001944601.2,APHIDBASE:ACYPI006159_ Name=XP_001944601.2	72509397	72509555	+	NC_042493.1	72509474	72509483	-1	reverse strand match		GACAAATCGC
Dbxref=GeneID:103310983,Genbank:XP_008188715.1_Name=XP_008188715.1	5796	6049	+	NW_021772934.1	5865	5874	1	plus plus match		GCGATTTGTC
Dbxref=GeneID:100169346,Genbank:NP_001155827.1,APHIDBASE:ACYPI009973_ Name=NP_001155827.1	147880420	147880596	+	NC_042494.1	147880500	147880509	-1	reverse strand match		GACAAATCGC
Dbxref=GeneID:100163372,Genbank:XP_016660060.1,APHIDBASE:ACYPI004465_ Name=XP_016660060.1	7829771	7829881	-	NC_042494.1	7829812	7829821	1	reverse complement match		GCGATTTGTC
Dbxref=GeneID:100162284,Genbank:XP_008184038.2,APHIDBASE:ACYPI003444_ Name=XP_008184038.2	42048682	42048888	+	NC_042494.1	42048750	42048759	-1	reverse strand match		GACAAATCGC
Dbxref=GeneID:100167175,Genbank:XP_016663116.1,APHIDBASE:ACYPI007989_ Name=XP_016663116.1	15289673	15289842	-	NC_042496.1	15289694	15289703	-1	plus plus match		GACAAATCGC
Dbxref=GeneID:100162939,Genbank:XM_001947352.4,APHIDBASE:ACYPI004058_ gbkey=mRNA	96558712	96559623	-	NC_042494.1	96559047	96559056	1	reverse complement match		GCGATTTGTC
Dbxref=GeneID:100158993,Genbank:XM_001951050.5,APHIDBASE:ACYPI000407_ gbkey=mRNA	60711788	60715374	-	NC_042494.1	60713165	60713174	-1	plus plus match		GACAAATCGC
Dbxref=GeneID:100159515,Genbank:XM_029487768.1,APHIDBASE:ACYPI000878_ gbkey=mRNA	91488174	91488582	-	NC_042494.1	91488434	91488443	1	reverse complement match		GCGATTTGTC

A.pisum_GCGATTTGTC_mm_match

Gene Name	CDS start position	CDS end position	Strand	Genome	Query start position	Query end position	Query strand	Comment	Extra Comment	Query sequence
Dbxref=GeneID:100575757,Genbank:XR_119073.4_gbkey=ncRNA	166874026	166874293	+	NC_042494.1	166874253	166874262	1	plus plus match		GCGATTTGTC
Dbxref=GeneID:100161496,Genbank:XM_001949241.5,APHIDBASE:ACYPI002714_gbkey=mRNA	80421827	80422946	+	NC_042493.1	80422502	80422511	-1	reverse strand match		GACAAATCGC
Dbxref=GeneID:100162079,Genbank:XP_029346544.1_Name=XP_029346544.1	88179820	88180157	-	NC_042495.1	88180101	88180110	1	reverse complement match		GCGATTTGTC
Dbxref=GeneID:100571342,Genbank:XM_016808574.2_gbkey=mRNA	29531497	29534399	-	NC_042495.1	29532923	29532932	1	reverse complement match		GCGATTTGTC
Dbxref=GeneID:100169346,Genbank:NM_001162355.2,APHIDBASE:ACYPI009973_gbkey=mRNA	147880420	147880926	+	NC_042494.1	147880500	147880509	-1	reverse strand match		GACAAATCGC
Dbxref=GeneID:100161423,Genbank:XM_029486894.1_gbkey=mRNA	28581886	28584363	-	NC_042494.1	28583271	28583280	-1	plus plus match		GACAAATCGC
Dbxref=GeneID:100169526,Genbank:XM_001945782.5,APHIDBASE:ACYPI010137_gbkey=mRNA	31146286	31146930	-	NC_042493.1	31146635	31146644	-1	plus plus match		GACAAATCGC
Dbxref=GeneID:100158993,Genbank:XP_001951085.1,APHIDBASE:ACYPI000407_Name=XP_001951085.1	60712567	60715140	-	NC_042494.1	60713165	60713174	-1	plus plus match		GACAAATCGC
Dbxref=GeneID:100573640,Genbank:XM_003246574.4_gbkey=mRNA	22936021	22936912	+	NC_042494.1	22936288	22936297	-1	reverse strand match		GACAAATCGC
Dbxref=GeneID:100169413,Genbank:NM_001162843.1,APHIDBASE:ACYPI010034_Note=The RefSeq transcript has 6 substitutions%2C 3 non-frameshifting indels compared to this genomic sequence	42010194	42010507	-	NC_042495.1	42010304	42010313	-1	plus plus match		GACAAATCGC
Dbxref=GeneID:100568756,Genbank:XP_003241676.1_Name=XP_003241676.1	109879234	109880057	-	NC_042494.1	109879646	109879655	-1	plus plus match		GACAAATCGC
Dbxref=GeneID:100571057,Genbank:XP_003247185.1_Name=XP_003247185.1	44394257	44394691	-	NC_042494.1	44394323	44394332	1	reverse complement match		GCGATTTGTC
Dbxref=GeneID:100572374,Genbank:XM_003247033.4_gbkey=mRNA	65905966	65907733	+	NC_042493.1	65906897	65906906	-1	reverse strand match		GACAAATCGC
Dbxref=GeneID:100573273,Genbank:XM_008186676.3_gbkey=mRNA	95964183	95964500	-	NC_042495.1	95964381	95964390	1	reverse complement match		GCGATTTGTC
Dbxref=GeneID:100574016,Genbank:XP_016663153.1_Name=XP_016663153.1	37039985	37040194	+	NC_042494.1	37040082	37040091	-1	reverse strand match		GACAAATCGC
Dbxref=GeneID:103308676,Genbank:XR_003839372.1_gbkey=misc_RNA	23501549	23503437	-	NC_042495.1	23503251	23503260	1	reverse complement match		GCGATTTGTC
Dbxref=GeneID:115033666,Genbank:XR_003838958.1_gbkey=ncRNA	19079630	19086527	+	NC_042494.1	19085940	19085949	-1	reverse strand match		GACAAATCGC
Dbxref=GeneID:100158860,Genbank:XM_001943185.5,APHIDBASE:ACYPI000281_gbkey=mRNA	23386362	23386751	-	NC_042495.1	23386663	23386672	-1	plus plus match		GACAAATCGC
Dbxref=GeneID:100572374,Genbank:XP_003247081.1_Name=XP_003247081.1	65906059	65907733	+	NC_042493.1	65906897	65906906	-1	reverse strand match		GACAAATCGC
Dbxref=GeneID:100168729,Genbank:XM_003240853.4,APHIDBASE:ACYPI009406_gbkey=mRNA	48022893	48023280	+	NC_042495.1	48023219	48023228	-1	reverse strand match		GACAAATCGC
Dbxref=GeneID:100573041,Genbank:XM_003243791.4_gbkey=mRNA	46328617	46330081	+	NC_042494.1	46329759	46329768	1	plus plus match		GCGATTTGTC
Dbxref=GeneID:107882420,Genbank:XR_003839664.1_gbkey=ncRNA	41653564	41653688	+	NC_042495.1	41653646	41653655	1	plus plus match		GCGATTTGTC
