# Supplementary material for: In vivo interference of pea aphid endosymbiont Buchnera groEL gene by synthetic peptide nucleic acids
Source: Sci Rep. 2024 Mar 5;14:5378. doi: 10.1038/s41598-024-55179-2 (PMC10912616; doi:10.1038/s41598-024-55179-2)
Supplement: Supplementary file 2 — Supplementary Information 2. [file 41598_2024_55179_MOESM2_ESM.pdf]

In Vivo Interference of Pea Aphid Endosymbiont _Buchnera groEL_ Gene by Synthetic Peptide Nucleic Acids

Kathrine Xin Yee TAN [1,2] and Shuji SHIGENOBU[1,2*]

[1]*Department of Basic Biology, School of Life Science, The Graduate University for Advanced Studies (SOKENDAI), 38 Nishigonaka, Myodaiji, Okazaki, Aichi 444-8585 Japan*

[2]*Laboratory of Evolutionary Genomics, National Institute for Basic Biology, Okazaki 444-8585, Japan*

*Correspondence:

Shuji Shigenobu

ORCID: 0000-0003-4640-2323

Laboratory of Evolutionary Genomics, National Institute for Basic Biology, 38 Nishigonaka Myodaiji, Okazaki, Aichi 444-0867, Japan

Phone: +81-564-55-7670; E-mail: shige@nibb.ac.jp

Kathrine Xin Yee Tan

ORCID: 0000-0002-8350-4719

E-mail: kathrine@nibb.ac.jp

Supplementary data

Table S1: List of primers used in qPCR and RT-qPCR analyses.

Primer	Sequence	Primer length (bp)	Amplicon length (bp)	Target gene	Efficiency (%)	Reference
BuchGroEL_1_F	AAACTATCAGGCGGTGTTGC	20	226	*groEL*	92.6	
BuchGroEL_R	CACGCAAAGCAACTCGAATA	20				
rpL7_F	GCGCGCCGAGGCTTAT	16	81	*rpl7*	100.0	[*]
rpL7_R	CCGGATTTCTTTGCATTTCTTG	22				
BuchPyrG_F	AATAGGCGGAACTGTTGGTG	20	248	*pyrG*	95.4	
BuchPyrG_R	TTTTTCGCTCGTGAAGAGGT	20				
BuchDnaK_F1018	GTTGGTGGTCAAACTAGAATGCCT	24	125	*dnaK*	101.8	
BuchDnaK_R1142	ACTCCTCCCTGTACTGCAGC	20				
Buch_rpoA_F	CAGGATGTGCGGTAACTGAA	20	185	*rpoA*		
Buch_rpoA_R	TGCGGCAGTAATAGAACCAA	20			98.7	
Buch_rrs_F274	AGGATAACCAGCCACACTGG	20	115			

Buch_rrs_R388	TCTTCATACACGCGGCATAG	20	16S rRNA gene	98.0

[*] Nakabachi, A., Shigenobu, S., Sakazume, N., Shiraki, T., Hayashizaki, Y., Carninci, P., ... & Fukatsu, T. (2005). Transcriptome analysis of the aphid bacteriocyte, the symbiotic host cell that harbors an endocellular mutualistic bacterium, *Buchnera*. *Proceedings of the National Academy of Sciences*, *102*(15), 5477-5482.

Table S2: Sequence matches of anti-*groEL* PNAs in *Buchnera aphidicola* str. APS genome.

Gene Name	CDS start position	CDS end position	Strand	Query start position	Query end position	Query strand	Comment	Extra Comment	Query sequence	
WP_009873980.1 (*groEL*)	18715	20361	+	NC_002528.1	18710	18719	-1	reverse complement match	Translational Start Site Included	GTCAAATGGC
WP_010896104.1 (*pyrG*)	451384	453021	+	NC_002528.1	451856	451865	-1	reverse complement match		GTCAAATGGC

Figure S1

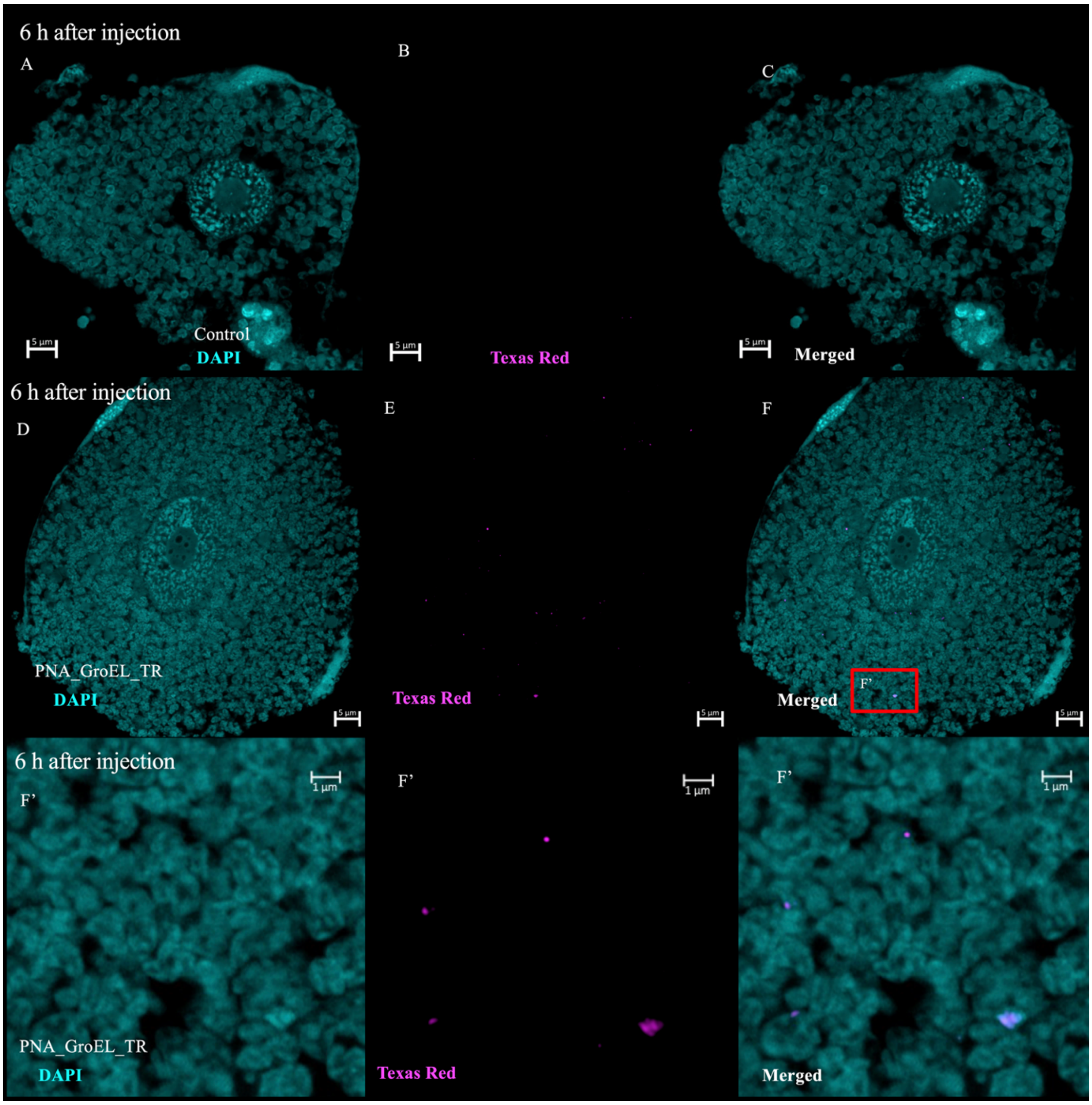

Figure S1: Super-resolution imaging of aphid bacteriocytes and *Buchnera* cells showed the signal of Texas Red which indicates the successful penetrated of PNA_GroEL (10 μM) into bacteriocytes 6 h after injection (D-F). $CaCl_2$ solution (12 mM) injected samples were used as the control in the experiment (A-C). Zoom-in image clearly showed the Texas Red signal and distorted *Buchnera* in the bacteriocytes of PNA_GroEL_TR treated sample (F').

Figure S2

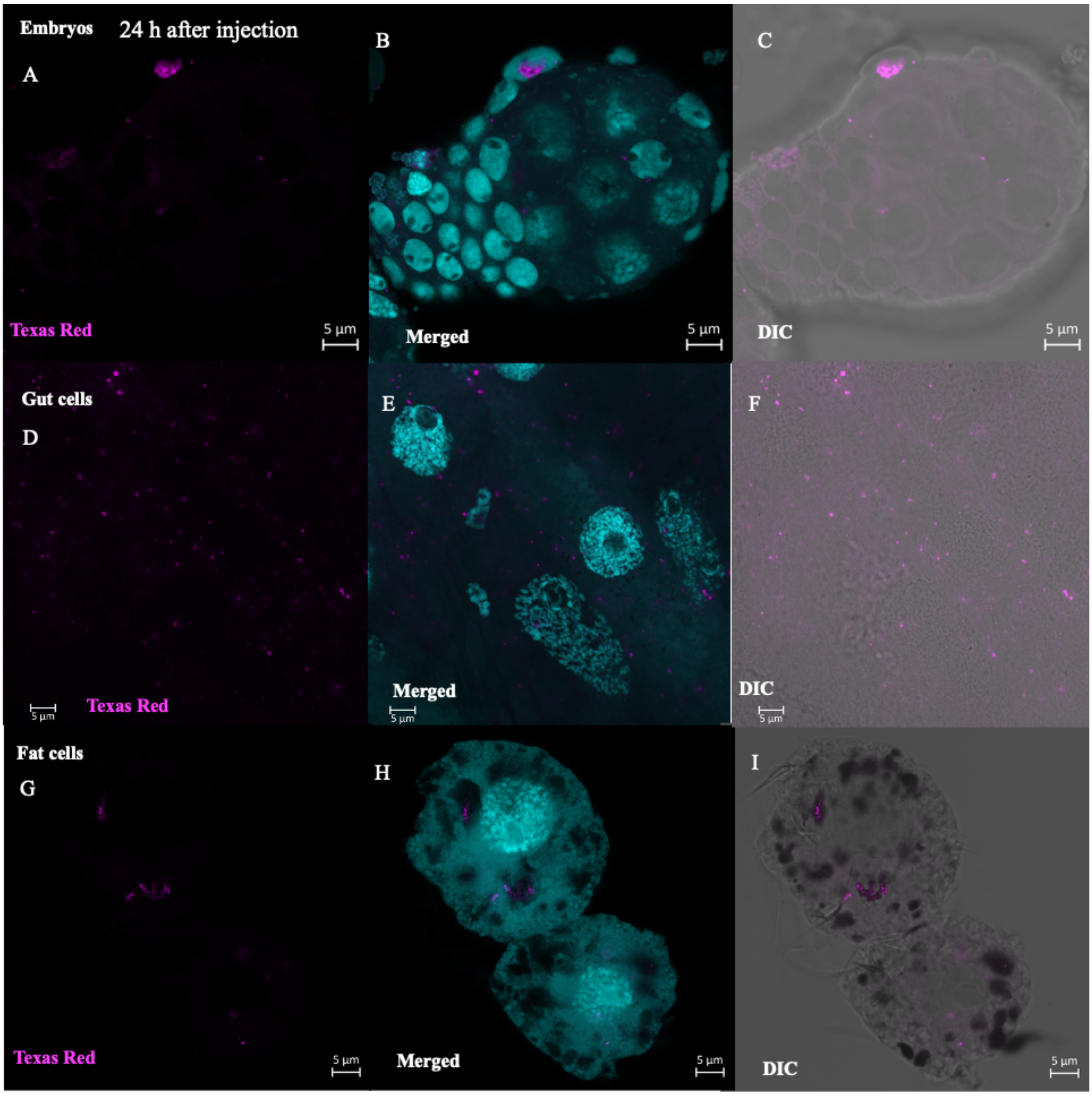

Figure S2: Super-resolution imaging of aphid bacteriocytes and *Buchnera* cells showed that the signal of Texas Red was detected in other aphid tissue such as fat cells, gut cells and embryos, in PNA_GroEL_TR (10 µM) treated samples (A-I). The images were taken using the aphid nymphs dissected after 24 h of PNA_GroEL_TR injection.

Figure S3

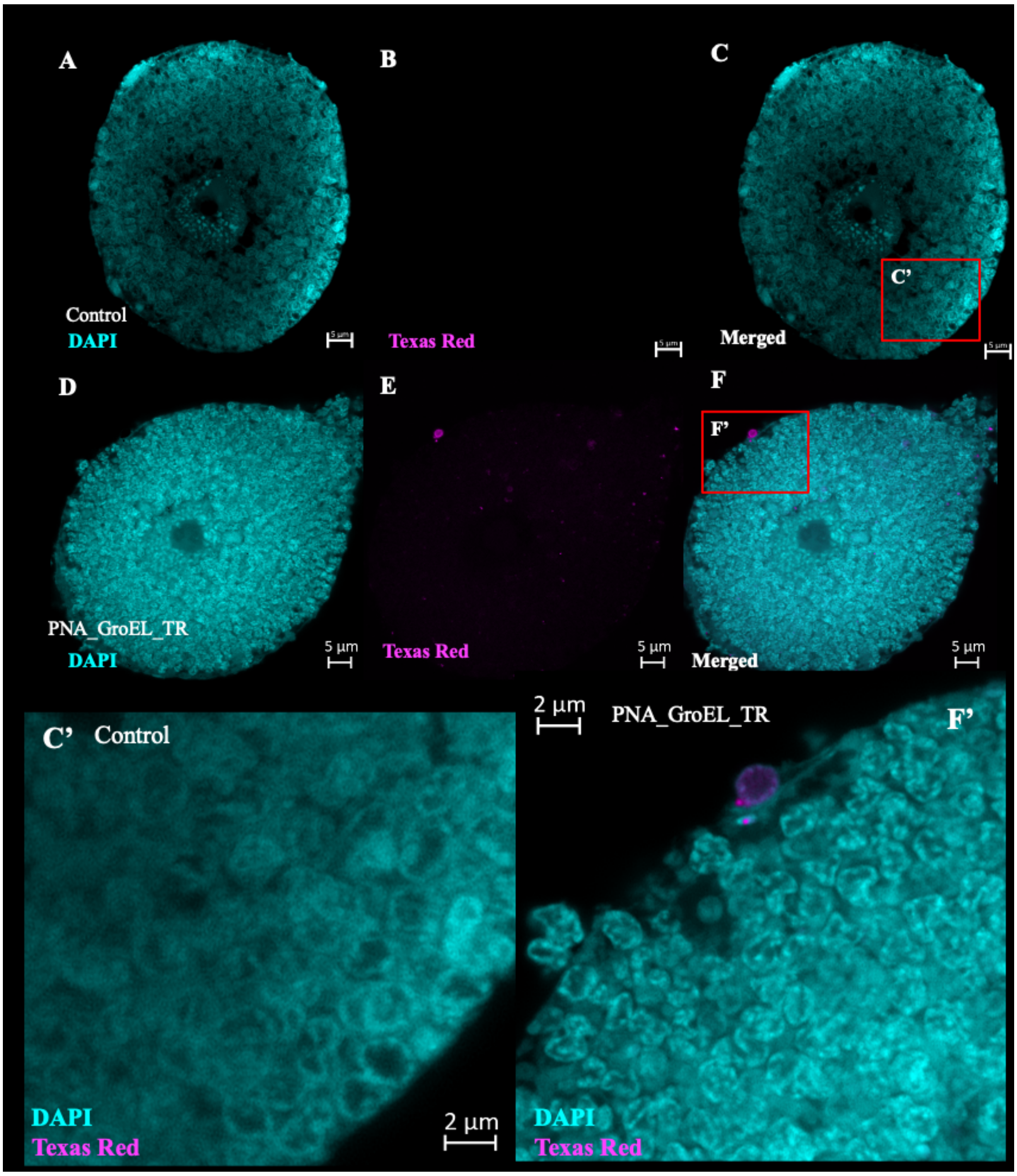

Figure S3: Super-resolution imaging of aphid bacteriocytes and *Buchnera* cells. Aphid nymphs were injected with $CaCl_2$ solution (12 mM) or PNA_GroEL_TR (10 µM in 12 mM $CaCl_2$ solution). Injected aphid nymphs were dissected after 24 h. Dissected bacteriocytes were fixed and stained with DAPI solution to mark the nuclei of bacteriocytes and chromosome of *Buchnera*. Aphid nymphs injected with $CaCl_2$ solution injected was used as the control (A-C). Detection of the Texas Red signal in bacteriocytes and *Buchnera* cells indicated the successful penetration of PNAs into the cells (D-F). Zoom-in images showed that the *Buchnera* cells of $CaCl_2$ treated aphid nymphs were mostly having a round shape (C') while distorted *Buchnera* cells were found in PNA_GroEL_TR treated sample (F').

Figure S4

Expression of *Buchnera rrs* and *rpoA* genes were not inhibited by the treatment of peptide-conjugated anti-*groEL* PNAs

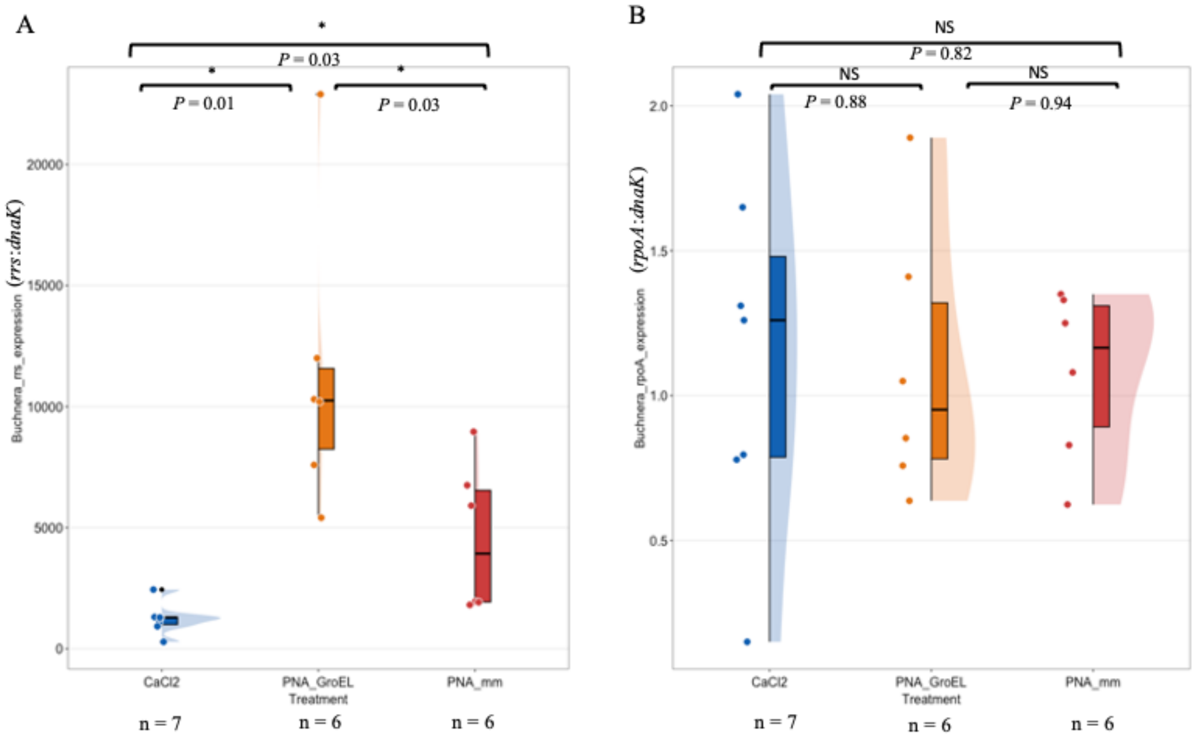

Figure S4: Comparison of *Buchnera rrs* and *rpoA* gene expression in PNAs injected aphid nymph. Second instar aphid nymphs were injected with 10 µM peptide-conjugated antisense *groEL* PNA (PNA_GroEL; orange plot) or control PNAs (PNA_mm; red plot). Additional negative control group, CaCl2 included aphid nymphs injected with 12 mM $CaCl_2$ solution (blue plot). A: A significant higher expression of *rrs* gene was found in the aphid nymphs treated with PNA_GroEL and PNA_mm than those with $CaCl_2$ solution. In comparison with PNA_mm, PNA_GroEL was also showing a significantly higher *rrs* gene expression. *P* values from pairwise comparisons using Wilcoxon rank-sum exact test are indicated on top of the graph. B: The difference in *Buchnera rpoA* gene expression between aphid nymphs treated with PNA_GroEL and PNA_mm was not significant. *P* values from Fisher's LSD post hoc test are indicated on top of the graph. Each black dot indicates a single PNAs treated aphid nymph.

To comprehensively investigate the impact of anti-*groEL* PNAs on untargeted gene without a perfect match to the design, we compared the expression of other *Buchnera* genes, i.e. *rrs* and *rpoA* between $CaCl_2$ solution, PNA_mm and PNA_GroEL treated aphid nymphs by RT-qPCR (Figure S4A). Meanwhile, the comparison of *Buchnera rpoA* gene expression between the

three treatments is shown in Figure S4B. The expression of *rrs* and *rpoA* genes were normalised by *Buchnera dnaK* gene expression. Kruskal-Wallis test shows that the median of CaCl2, PNA_mm and PNA_GroEL were significantly different from each other ($H(2) = 13.41$, $p = 0.001$). *Buchnera rrs* gene expression in aphid nymphs treated with 10 µM PNA_GroEL (*Mdn* = 10250, *SD* = 6093.54) and PNA_mm (*Mdn* = 3925, *SD* = 3078.13) was significantly higher than those in CaCl2 (*M* = 1290, *SD* = 644.61) ($p = 0.01$ for PNA_GroEL; $p = 0.03$ for PNA_mm). In addition, PNA_GroEL was also showing a significant higher *rrs* gene expression compared to PNA_mm-treated aphid nymphs ($p = 0.03$). On the other hand, no significant difference was detected in the expression of the *rpoA* gene between CaCl2 (*M* = 1.14, *SD* = 0.62), PNA_GroEL (*M* = 1.10, *SD* = 0.47), PNA_mm (*M* = 1.08, *SD* = 0.30) (ANOVA, $F(2,16) = 0.03$, $p = 0.97$).

Access link of Google Colab notebook for finding the sequence match of PNAs:
https://colab.research.google.com/drive/1J8flkB8qwORqKCCLrDnBlOxKlVGtP1uF?usp=sharing

The code and the result tables are also available as Supplementary Code.
